# ﻿*Endiandramacrocarpa* (Lauraceae), a new tree species from south-western China

**DOI:** 10.3897/phytokeys.224.102752

**Published:** 2023-04-07

**Authors:** Dian-yang Zou, Guan-long Cao, Jin-guo Zhang, Lang Li, Jie Li

**Affiliations:** 1 Plant Phylogenetics and Conservation Group, Center for Integrative Conservation & Yunnan Key Laboratory for Conservation of Tropical Rainforests and Asian Elephants, Xishuangbanna Tropical Botanical Garden, Chinese Academy of Sciences, Mengla, Yunnan 666303, China University of Chinese Academy of Sciences Beijing China; 2 University of Chinese Academy of Sciences, Beijing 100049, China Xishuangbanna Tropical Botanical Garden, Chinese Academy of Sciences Mengla China; 3 State Key Laboratory of Systematic and Evolutionary Botany, Institute of Botany, Chinese Academy of Sciences, Beijing 100093, China Institute of Botany, Chinese Academy of Sciences Beijing China; 4 Administration Bureau of Maguan Gulinqing Provincial Nature Reserve, Wenshan, Yunnan 663000, China Administration Bureau of Maguan Gulinqing Provincial Nature Reserve Wenshan China

**Keywords:** *Endiandra*, morphology, taxonomy, tropical montane forest, Yunnan Province

## Abstract

*Endiandramacrocarpa*, a new species of *Endiandra* (Lauraceae) from Yunnan Province of south-western China, is here described and illustrated, based on morphological evidence. Compared to other *Endiandra* species occurring in south China and the adjacent regions in Indochina, this species is mainly characterised by its much larger ellipsoidal fruits (up to 11 × 6 cm), as well as glabrous branchlets and puberulent inflorescences.

## ﻿Introduction

The genus *Endiandra* R. Br. of the Lauraceae family is widely distributed from south China, Indochina, Malesia and Australia to the Pacific Islands (Rohwer 1993; Arifiani 2001; van der Werff 2001). It has approximately 100 species and its diversity is strongly centred in south-eastern Malesia and Australia (Hyland 1989; Arifiani 2001; Cussan et al. 2007). *Endiandra* was first described by Brown (1810), based on the type species from Australia, *Endiandraglauca*. The species of the genus can be characterised by alternate, penninerved leaves; axillary or terminal panicles; bisexual flowers with three 2-celled fertile stamens and unprotected fruits on pedicels (Kostermans 1957; Rohwer 1993; van der Werff 2001; Cussan et al. 2007; Li et al. 2008).

According to previous studies of wood and bark anatomy, floral morphology, taxonomy and molecular phylogeny, *Endiandra* belongs to the basal lineages of the family, in the tribe Cryptocaryeae or the *Cryptocarya* group and is closely related to *Beilschmiedia* Nees (Richter 1981; Rohwer 1993; van der Werff and Richter 1996; Rohwer 2000; Chanderbali et al. 2001; Rohwer and Rudolph 2005; Rohwer et al. 2014; Li et al. 2020; Song et al. 2020). Vegetatively, *Endiandra* is very similar to *Beilschmiedia*, only flower characters can differentiate the two genera (van der Werff 2001; Arifiani et al. 2012). Typical flowers of *Endiandra* only have three fertile stamens in the third whorl, whereas *Beilschmiedia* has nine fertile stamens (Arifiani 2001; van der Werff 2001; Arifiani et al. 2012).

Without any comprehensive revision, *Endiandra* has so far only been treated in floras or local revisions (e.g. Hyland (1989); Kochummen (1989); Arifiani (2001); Cussan et al. (2007); Li et al. (2008)). In China, there are only three recognised *Endiandra* species (two endemic) and they are distributed in Yunnan, Guangxi, Hainan and Taiwan (Li et al. 1979; Li et al. 1982; Liang et al. 1985; Li et al. 2008; Yang and Da 2008; Yu et al. 2009). During recent field surveys in south-eastern Yunnan Province, we collected an unknown Lauraceae species with very large fruits. Further morphological study suggests that this species belongs to *Endiandra* and differs from its other species distributed in south China and the adjacent regions. As a result, we here describe this species as new to science.

## ﻿Materials and methods

We conducted field surveys from 2020 to 2022. Morphological characters of the new *Endiandra* species were examined in detail, based on fresh and preserved materials, as well as dried specimens. We also compared the new species with possible relatives, based on specimens from the herbaria HITBC, IBK, IBSC, KUN, PE, SYS and SZ and images of specimens available on JSTOR Global Plants (www.plants.jstor.org) and GBIF (www.gbif.org).

## ﻿Results

### ﻿Taxonomic treatment

#### ﻿ Endiandra macrocarpa

Taxon classificationPlantaeLauralesLauraceae

D.Y.Zou, Lang Li & J.Li sp. nov.

26B9495D-CE41-5454-8943-80B415741874

urn:lsid:ipni.org:names:77317221-1

Figs 1 , 2

##### Diagnosis.

Compared to other *Endiandra* species occurring in south China and the adjacent regions in Indochina, this species is mainly characterised by its much larger ellipsoidal fruits (up to 11 × 6 cm), as well as glabrous branchlets and puberulent inflorescences.

##### Type.

China. Yunnan Province: Maguan County, Gulinqing Town, Houcao Village, in tropical montane forest near the village and is strongly disturbed by human activities, ca. 800 m a.s.l., 12 May 2022, flowering, *Lang Li and Dian-yang Zou, 2022028* (Holotype: HITBC!; Isotypes: HITBC!).

##### Description.

**Figure 1.**
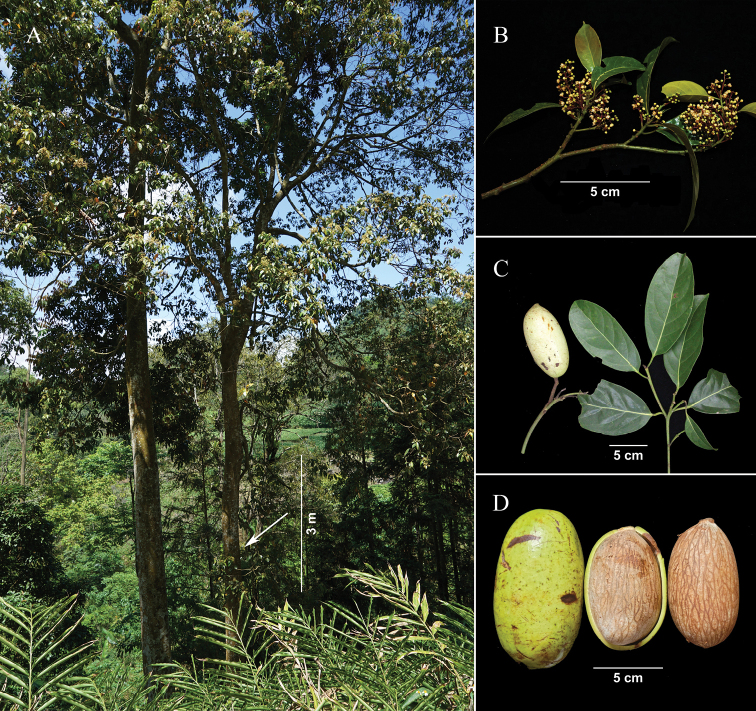
Morphology of *Endiandramacrocarpa***A** tree habit **B** flowering branchlet **C** fruiting branchlet displaying immature fruit **D** mature fruits. Photographed by Lang Li and Guan-long Cao.

**Figure 2.**
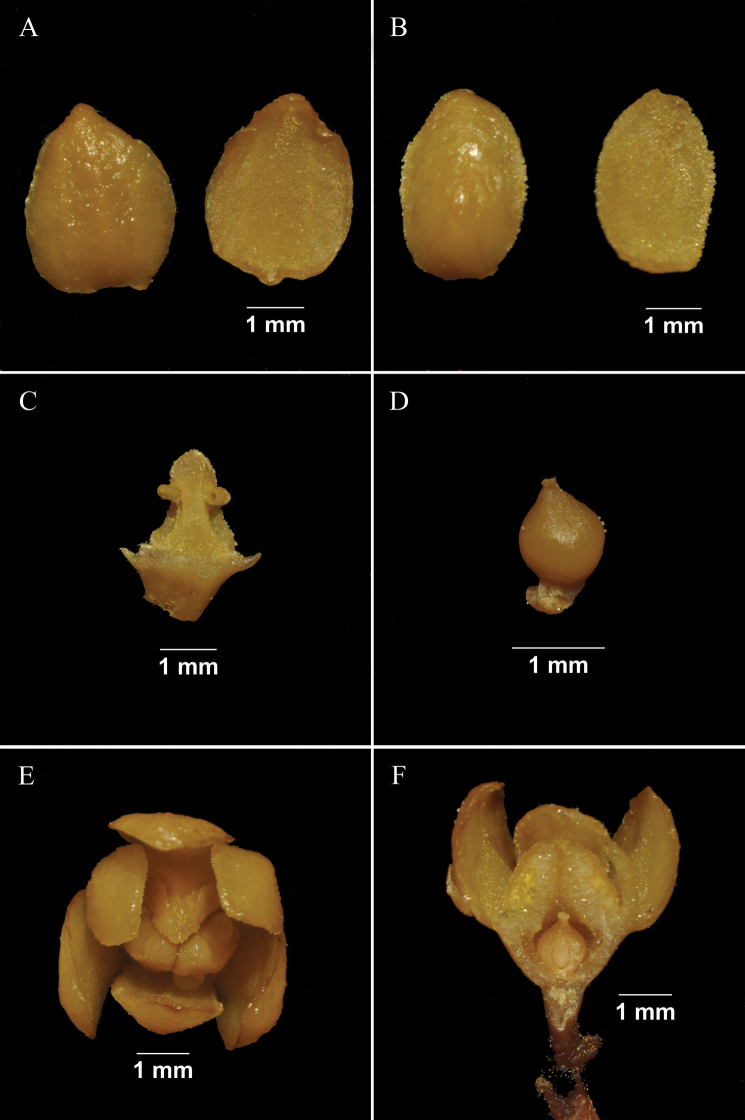
Morphology of a flower of *Endiandramacrocarpa*. **A** outer tepals, abaxial and adaxial side **B** inner tepals, abaxial and adaxial side **C** stamen, abaxial side **D** pistil **E** flower, top view **F** flower, longitudinal section. Photographed by Dian-yang Zou.

Trees evergreen, up to 15 m tall (Fig. 1A). Bark brownish-grey. Branchlets brown, terete, with blunt ridges and striate when dry, glabrous, slightly warty. Leaves alternate; petiole 1–1.5 cm, concave-convex, glabrous; leaf blade greenish and opaque abaxially, green and shiny adaxially, elliptic or oblong-elliptic, 5–16 × 3–7 cm, thinly leathery. Mid-rib elevated on both surfaces, but rather conspicuous abaxially, lateral veins 5–8 pairs, slightly elevated abaxially, conspicuous adaxially, veins and veinlets reticulate, base cuneate to obtuse, mostly asymmetric, apex acuminate with obtuse acumen or obtuse with acute acumen. Panicle axillary, 4–8(10) cm, puberulent (Fig. 1B). Pedicels slender, 1–3 mm, thickened after anthesis. Flowers yellow, scented, ca. 3 mm. Perianth fleshy, unequal, outer ones slightly larger, broadly ovate, 3 × 2 mm (Fig. 2A), adaxially pilose; inner ones smaller, ovate, 2.5 × 1.8 mm, adaxially densely villous (Fig. 2B). Fertile stamens 3, triangular, ca. 2 mm, eglandular, puberulent; anthers thick, stalkless, 2-celled, cells extrorse, tightly adnate to each other; staminodes absent (Fig. 2C, E). Ovary ovoid, ca. 1.2 mm; style short; stigma punctate (Fig. 2D). Fruit ellipsoid or long ellipsoid, up to 11 × 6 cm, immature fruit green, yellow when mature, smooth, glabrous, apex bluntly apiculate (Fig. 1C, D). Seed endocarp light brown with a darker brown network of both broad and fine, slightly raised veins. Fruit stalk brown, up to 5 mm in diam. at apex, glabrous.

##### Phenology.

Flowering from April to May and fruiting from July to October.

##### Distribution and habitat.

**Figure 3.**
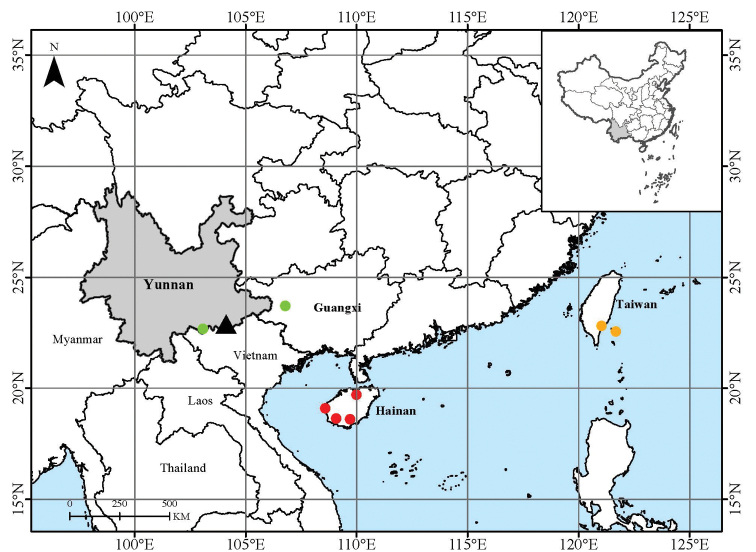
Distribution of *E.macrocarpa* (black triangle), *E.hainanensis* (red dot), *E.dolichocarpa* (green dot) and *E.coriacea* (yellow dot) in China.

Currently known only from the type locality in Maguan County, Yunnan Province, south-western China (Fig. 3). Tropical montane forest in valley, on clay loam soil mixed limestone; ca. 800 m a.s.l.

##### Etymology.

The specific epithet “*macrocarpa*” of the new species refers to its very large fruits compared to the other species within the genus *Endiandra*.

##### Preliminary conservation status.

Currently, *E.macrocarpa* is only known from the type locality in Maguan County, Yunnan Province, south-western China with only one mature individual. It is located in tropical montane forest near the village, which is strongly disturbed by human activities. So far, no other occurrence of this species in south-eastern Yunnan and the adjacent regions has been found or reported. Further in-depth field surveys are suggested in order to find more individuals and locations of the species. Thus, the preliminary conservation status of *E.macrocarpa* is not assessed in the present study.

##### Additional specimen examined.

China. Yunnan Province: Maguan County, Gulinqing Town, Houcao Village, in tropical montane forest near the village, strongly disturbed by human activities, ca. 800 m a.s.l., 26 August 2020, fruiting, *Lang Li and Guan-long Cao, 2020158* (HITBC!); 25 April 2021, flowering, *Lang Li and Guan-long Cao, 2021029* (HITBC!); 28 October 2021, mature fruits, *Lang Li and Dian-yang Zou, 2021081* (HITBC!).

## ﻿Discussion

South-eastern Yunnan is biogeographically located in a transitional zone from tropical Southeast Asia to subtropical East Asia (Zhu and Yan 2009). The flora of this region is suggested to be a marginal part of the tropical Asian flora, but contains more subtropical and temperate elements than other floras of the adjacent regions, for example, southern Yunnan, south-western Guangxi and northern Vietnam (Zhu and Yan 2009; Zhu 2013). As one of the world’s biodiversity hotspots, south-eastern Yunnan is of extreme interest to botanists not only for its richness of primitive angiosperm taxa, such as species of Lauraceae and Magnoliaceae, but also for being a centre of plant endemism in China (Myers et al. 2000; Zhu and Yan 2009; López-Pujol et al. 2011). A recent study conducted by Zhou et al. (2023) further revealed that south-eastern Yunnan is an endemism centre of Lauraceae with significantly high species richness (SR), phylogenetic diversity (PD), corrected weighted endemism (CWE) and phylogenetic endemism (PE). Recent field surveys in this region also discovered several new endemic species of Lauraceae, for example, *Beilschmiediaturbinata* Bing Liu & Y. Yang, *Caryodaphnopsismalipoensi*s Bing Liu & Y. Yang, *Phoebehekouensis* Bing Liu, W.Y. Jin, L.N. Zhao & Y. Yang and *Phoebejinpingensis* Bing Liu, Y. Yang, W.Y.Jin & Zhi Yang (Liu et al. 2013a, b, 2020; Yang et al. 2021).

Far away from its diversity centre in the south-eastern part of Malesia and Australia, *Endiandra* species are very rare in China. Besides the newly-discovered *E.macrocarpa*, only three recognised *Endiandra* species (two endemic) are distributed in Yunnan, Guangxi, Hainan and Taiwan (Li et al. 2008, Fig. 3). Considering the possible endemism of the new species, we firstly compared *E.macrocarpa* with the other three *Endiandra* species occurring in China, which possess much smaller fruits (detailed in Table 1). Fruits of *E.coriacea* Merr. are ovoid, up to 2 × 1 cm. Fruits of *E.hainanensis* Merr. et F.P. Metcalf ex Allen are narrowly ellipsoid, up to 3.8 × 1.4 cm. Fruits of *E.dolichocarpa* S. Lee et Y. T. Wei are cylindrical and larger, up to 8 × 2.3 cm, but still much smaller than the fruits of *E.macrocarpa*. Additionally, *E.macrocarpa* has glabrous branchlets and puberulent inflorescences, while twigs are puberulent in *E.coriacea* and panicles are glabrous in *E.hainanensis* (Li et al. 2008). We also compared *E.macrocarpa* with three other *Endiandra* species occurring in the adjacent regions of south-eastern Yunnan in Indochina, for example, Vietnam, Laos and Thailand (detailed in Table 1). *Endiandrafirma* Nees differs from *E.macrocarpa* by its smaller fruits with rounded tips (Hooker 1875). *Endiandramacrophylla* (Blume) Boerl. differs from *E.macrocarpa* by its smaller fruits and much larger leaves (Arifiani 2001). *Endiandrarubescens* Blume ex Miq. differs from *E.macrocarpa* by its smaller fruits and pubescent branchlets (Arifiani 2001). Species with giant fruits are uncommon in *Endiandra*. *Endiandrainsignis* (F.M.Byailey) F.M.Byailey and *E.sulavesiana* Kosterm., endemic to Australia and Sulawesi, respectively, are the two *Endiandra* species that possess fruits with comparable size to those of *E.macrocarpa* (Kostermans 1955; Cussan et al. 2007). However, other fruit characters of these two species are quite different. *Endiandrainsignis* has globular, ribbed fruits (6–8 × 6.5–10.1 cm) and *E.sulavesiana* has long cylindrical, ribbed fruits (up to 13 × 2.5 cm), while *E.macrocarpa* has ellipsoidal, smooth fruits (up to 11 × 6 cm). As a result, morphological evidence supports the recognition of *E.macrocarpa* as a distinct species in the genus *Endiandra*.

**Table 1.** Comparative morphology, habitat and geographic distribution of *Endiandramacrocarpa* and its possible relatives.

Morphological character	*Endiandramacrocarpa*	*Endiandrahainanensis*	*Endiandradolichocarpa*	*Endiandracoriacea*	*Endiandrafirma*	*Endiandramacrophylla*	*Endiandrarubescens*
**Leaf**	elliptic or oblong-elliptic, 5–16 × 3–7 cm, thinly leathery, lateral veins 5–8 pairs, petiole 1–1.5 cm, glabrous	lanceolate to oblong-elliptic, 9–15 × 3–6 cm, papery, lateral veins 6–8 pairs, petiole 1–1.5 cm, glabrous	oblong, 13–25(30) × (4)5–7.5(11) cm, leathery, lateral veins 6–8 pairs, petiole robust, up to 2 cm, glabrous	elliptic or obovate, 9–12 × 4.5–6 cm, thickly leathery, lateral veins 5 or 6 pairs, petiole 1–1.2 cm, puberulent initially, but soon glabrate	oblong-elliptic, 15–20 × 4 cm, glabrous, lateral veins 10–11 pairs, petiole 1.2 cm	elliptic to slightly obovate, 16–30 × 5–13 cm, lateral veins 8–13 pairs, petiole 1.2–2.5 cm, glabrous	elliptic, 6.5–15 × 2–7 cm, lateral veins 7–11 pairs, petiole 0.8–1.5 cm, nearly glabrous
**Branchlet**	glabrous	glabrous	glabrous	puberulent	–	glabrous	pubescent
**Fruit**	ellipsoid or long ellipsoid, up to 11 × 6 cm, yellow when mature, glabrous, apex bluntly apiculate	narrowly ellipsoid, up to 3.8 × 1.4 cm, purple-brown when mature, glabrous, obtuse at both ends	cylindrical when dry, up to 8 × 2.3 cm, black-brown when mature, glabrous, obtuse on both ends	ovoid, up to 2 × 1 cm, glabrous, base subrounded, apex acute	elliptic-ovoid, up to 6.4 cm long, quite smooth, tip rounded	ellipsoid, 4–7.5 × 1.7–2.5 cm, base obtuse	ellipsoid, green, 2–5 × 1.3–2.5 cm, base obtuse
**Inflorescence**	axillary, 4–8(10) cm, puberulent	axillary, 2–6 cm, few flowered, glabrate	–	axillary or terminal, up to 8 cm, few flowered, puberulent	2.5–5 cm, obscurely puberulent	axillary, 6–15 cm, with a sparse or dense, short, erect indument	axillary, 4–13 cm, with a sparse or dense, short, erect indument
**Habitat**	tropical montane forest in valley, on clay loam soil mixed limestone; ca. 800 m	mixed forests in valleys, thickets on open land; ca. 400–1100 m	forests; ca. 500 m.	low hill forests; ca. 20–200 m	montane rain forest on sandy loam soil; ca.100–1800 m	primary rain forest or peat swamp-forest on clay loam soil or sandy soil; ca. 50–1100 m	primary rain forest on sandy loam or acid soils along streams; ca. 20–1500 m
**Distribution**	SW China (Yunnan)	S China (Hainan)	SW China (Guangxi, Yunnan)	SE China (Taiwan), Philippines	Bangladesh, India, Vietnam, Malaysia, Indonesia	Thailand,Vietnam, Laos, Malaysia, Singapore, Philippines, Indonesia	Vietnam, Malaysia, Indonesia, Papua New Guinea, Australia

Note: “–” represents unknown morphological characters.

## Supplementary Material

XML Treatment for Endiandra macrocarpa
